# Community acceptability of dolutegravir-based HIV treatment in women: a qualitative study in South Africa and Uganda

**DOI:** 10.1186/s12889-020-09991-w

**Published:** 2020-12-07

**Authors:** Yussif Alhassan, Adelline Twimukye, Thoko Malaba, Catherine Orrell, Landon Myer, Catriona Waitt, Mohammed Lamorde, Andrew Kambugu, Helen Reynolds, Saye Khoo, Miriam Taegtmeyer

**Affiliations:** 1 Community Health Systems Group, Department of International Public Health, Liverpool School of Tropical Medicine, Pembroke Place, Liverpool, L3 5QA UK; 2 Infectious Disease Institute, Kampala, Uganda; 3 School of Public Health and Family Medicine, University of Cape Town, Cape Town, South Africa; 4 Desmond Tutu HIV Centre, Cape Town, South Africa; 5 Institute of Systems, Molecular and Integrative Biology, University of Liverpool, Liverpool, UK; 6 Tropical Infectious Diseases Unit, Royal Liverpool University Hospital, Prescot Street, Liverpool, UK

**Keywords:** Acceptability, Dolutegravir-based regimen, HIV treatment, Qualitative study, Uganda, South Africa

## Abstract

**Background:**

Despite concerns about dolutegravir use in pregnancy, most low- and middle-income countries are accelerating the introduction of dolutegravir-based regimens into national antiretroviral treatment programmes. Questions remain about the acceptability of dolutegravir use in women due to the potential risks in pregnancy. This study from South Africa and Uganda explored community values, preferences and attitudes towards the use of dolutegravir-based regimens in women.

**Methods:**

This study employed a qualitative design involving in-depth interviews and focus group discussion conducted between August 2018 to March 2019. The study was conducted in the months following an announcement of a potential risk for neural tube defects with dolutegravir use among women during conception and the first trimester. Participants included HIV positive pregnant and lactating women and their partners. They were selected purposively from urban poor communities in South Africa and Uganda. Data was analysed thematically in NVivo.

**Results:**

Forty-four in-depth interviews and 15 focus group discussions were conducted. Most participants had positive views of dolutegravir-based regimens and perceived it to be more desirable compared with efavirenz-containing regimens. There was widespread concern about use of dolutegravir during pregnancy and among women of childbearing age due to publicity around the possible association with neural tube defects. Acceptability was gendered, with nearly all male participants preferring their female spouses of childbearing potential not to use dolutegravir, while most women not planning pregnancy wanted access to contraception alongside dolutegravir. Community awareness and knowledge of dolutegravir was low and characterised by negative information. Women were concerned about HIV-related stigma and wanted the privacy features of dolutegravir to be strengthened with modification of the pill appearance and disguised packaging.

**Conclusions:**

Dolutegravir-based regimens were found to be generally acceptable for use in women except during pregnancy. Interest in a dolutegravir-based regimen was linked with its perceived potential to enhance health, privacy and reduce stigma while concerns about neural tube defects were the main potential barrier to dolutegravir uptake in women. In order to optimise the community acceptability and uptake of acceptability-based regimen among women it is critical to strengthen community awareness and understanding of dolutegravir treatment, improve contraception services alongside the introduction of dolutegravir, and engage with male partners.

**Supplementary Information:**

The online version contains supplementary material available at 10.1186/s12889-020-09991-w.

## Background

Since 2016 many low– and middle -income countries (LMICs) have accelerated the transition to dolutegravir-based first-line HIV treatment regimens and away from non-nucleoside reverse-transcriptase inhibitors (NNRTIs) such as efavirenz and nevirapine. The World Health Organisation (WHO), in guidelines released in 2016 [1] and 2018 [2], encouraged countries with levels of pre-treatment resistance to NNRTIs above 10% to consider switching to an alternative antiretroviral (ARV) drug [3]. Dolutegravir is linked with greater viral suppression than efavirenz [2], lower risks of drug-drug interactions [4] and lower risk of emergence of drug resistance mutations [5–7]. Programmatically, large-scale rollout of dolutegravir-based regimen means increased ART harmonisation, simplified drug procurement and lowering of ART costs [2]. Recent modelling studies indicate overall public health benefits from wider dolutegravir use in terms of reduced deaths among women, decreased HIV transmission and disability-adjusted life years [8, 9].

In May 2018, preliminary results from the Tsepamo study in Botswana indicated a higher risk for neural tube defects (NTDs) in infants born to women living with HIV who conceived while taking dolutegravir when compared to infants born to women receiving efavirenz (4 in 426 infants; 0.94% vs 3 in 5787 infants; 0.05%) [10, 11]. Following this, the WHO released interim guidelines recommending a cautious approach to dolutegravir use in women peri-conception [12]. Dolutegravir use was discouraged unless women of reproductive potential were using effective contraception or already in the second or third trimesters of pregnancy [2]. In July 2019, WHO guidance was updated to recommend dolutegravir as the preferred first-line regimen for all women provided they had received sufficient information on potential risks, benefits and contraceptive options to make an informed choice [13]. Revised recommendations were informed by updated data from the Tsepamo study which revealed a substantially lower, but still not insignificant, risk for NTDs than originally reported (0.3%) [14].

By mid-2019, 123 LMICs had either adopted or were planning to switch to dolutegravir-based regimens for first line treatment in their national ART programmes [15]. It is forecasted that by 2021 approximately 15 million people would be using dolutegravir-based regimens with efavirenz-containing regimens being replaced [16]. In Uganda, after a pilot in late 2017, the national roll-out of dolutegravir-based regimens commenced in March 2018 with access initially restricted among women of childbearing potential but later extended to include all adults in line with new WHO guidelines [17]. In the yet to be implemented South African guidelines published in October 2019, dolutegravir is recommended as the preferred regimen for all adults except in women around the time of conception and in the first trimester of pregnancy. Women of childbearing potential are encouraged to use contraception if taking dolutegravir [18].

As LMICs roll-out dolutegravir-based regimens in their HIV treatment programmes, a key question is how communities perceive the risks and benefits of the transition. The potential risks of dolutegravir in pregnancy could profoundly influence community acceptability and attitudes towards its use in women of childbearing age [19]. Women may lack accessible information and services and may face difficulties in decision-making despite WHO guidelines emphasising individual choice. Published studies are predominantly survey-based and provide limited data to predict community behaviour [2, 16]. Since dolutegravir is new with potential risks of use in pregnancy, information on the values, preferences and attitude of women and their male partners towards the drug would be useful in informing appropriate program and policy decisions to facilitate rollout among women.

We set out to explore community acceptability of dolutegravir use in women, including potential barriers to uptake related to community understandings, to knowledge of side effects in pregnancy and to the attitudes, values and preferences of women and their male partners. This qualitative study was a component of DolPHIN-2, a randomised controlled trial (NCT03249181) conducted in Uganda and South Africa that found that dolutegravir is well-tolerated and achieves superior virological suppression compared to efavirenz (the standard first-line HIV drug for pregnant women in both countries) when initiated in women presenting late in pregnancy [20].

## Methods

### Study design

A descriptive qualitative design was used to better understand participants’ perspectives on the acceptability of the use of dolutegravir-based treatment among women (including attitudes, preferences and values about the drug) and the context in which those perspectives are situated [21]. In-depth-interviews (IDIs) and focus group discussions (FGDs) were used to gain insights into individual and community perspectives for data triangulation purposes [22]. Data collection was staggered to allow for key findings discovered in the IDIs to be explored further and validated in the FGDs. The Standards for Reporting Qualitative Research were employed to prepare this manuscript to ensure greater transparency [23].

### Study settings

In South Africa, participants were recruited from Gugulethu, a peri-urban township in Western Cape with approximately 98,500 people [24]. The area is economically deprived with a high level of HIV among women, estimated at 18.9% in 2015 [25]. Participants in Uganda were selected from poor urban communities in and around Kampala, with HIV prevalence of around 6.9% [26]. Both settings had high background rates of violence against women and girls, which may have contributed to a disproportionately higher HIV infection among women [27, 28].

### Study population and participant selection

The study population included HIV-positive pregnant or lactating women 18 years and older and their partners. We included DolPHIN-2 trial participants initiated on dolutegravir-based ART at presentation in the third trimester of pregnancy. In Uganda, we also included non-trial participants who had previously used dolutegravir after participating in the pilot programme. The dolutegravir-experienced participants were needed to better understand their experience of the drug. Women who were still on efavirenz regimen and had never used dolutegravir, were included to learn about their understanding of the new treatment and male partner perspectives were included to help understand treatment decision making in patriarchal societies [29].

Purposive sampling approach was applied to select the study participants [30]. They were selected from DolPHIN-2 affiliated health facilities, which provided antenatal and postnatal care and/or ART services, including the Infectious Disease Institute and the Kasangati Health Centre in Uganda and Gugulethu Maternity and Obstetric Unit in South Africa. Health workers identified eligible women and referred them to research assistants (RAs) who used a participant screening checklist to verify eligibility. Eligible women were invited to participate in the study; they chose which data collection method they preferred (IDI or FGD); and a suitable date and venue was agreed. All the IDIs and FGDs were conducted in convenient and safe locations within the facilities and community centres where the participants were recruited.

An initial quota of 15 IDIs with women and 3 FGDs with men and women in each study country were initially set with actual sample size determined by data saturation. Participants were recruited and data collected until all aspects of the research question were sufficiently explored and new data were redundant [31]. Data saturation was recognised through daily debriefing involving the wider research team. No further participant recruitment was needed after the data were analysed.

### Data collection

Data were collected by RAs between August 2018 – March 2019. Both male and female RAs experienced in qualitative data collection were selected from the local community as they spoke the local languages and understood the social and cultural contexts of the study. Selected RAs were given further training in the study protocol and research ethics, including confidentiality and consent administration. Data collection was carried out around the time of the release of the initial results of the Tsepamo study [21] and the WHO safety alert [22], which may have shaped some participants’ awareness and views on the subject as well as their willingness to participate in the study. Neither study country had started rolling out dolutegravir-based regimens in the wider health system at the time of data collection.

Data collection was staggered to allow for emerging results from IDIs to be validated during subsequent FGDs. IDIs were carried out with women as they were the primary respondents for the study. FGDs were conducted with both men and women and were organised separately for the different genders so that participants could share their views freely. The IDIs and FGDs involving women were carried out by female RAs. Each FGD included 6–12 participants and was conducted by two RAs, with one moderating and the other taking notes. Interviews were conducted in Xhosa in South Africa and Luganda in Uganda in safe and private locations within facilities (IDIs) and community centres (FGDs). Each IDI and FGD lasted for approximately 1 h, was audio-recorded and complemented by written notes. Topic guides were developed based on Sekhon et al.’s framework of acceptability [32] and the WHO 2018 ART guidelines [2], and included knowledge and awareness, perceived benefits and risks, barriers to uptake of and attitudes towards dolutegravir-based treatment. As the study countries had not yet revised their guidelines, hypothetical scenarios were incorporated into the topic guides to give participants a sense of how dolutegravir may be used among women of childbearing potential. These were intended to encourage participants to think deeply and accurately about the topic rather than obtain quantifiable results. Topic guides were piloted and revised iteratively as the data collection evolved. The daily debriefings allowed for regular review and revision of the topic guides.

### Data analysis

Audio recordings were transcribed verbatim and translated into English by independent professionals. Data verification for accuracy and completeness was done through review of the transcripts by the RAs. Data were analysed in NVivo 12 software based on a thematic approach [33]. A common coding framework was inductively developed to facilitate inter-country comparison of the data. In order to do this, 10% of the transcripts from each context were initially reviewed by two researchers (YA and AT) for recurring themes to develop independent coding lists; these were compared and merged; consensus was sought for variations in coding [34]. The framework was developed with flexibility to accommodate emergent new themes as coding evolved. Using the framework, each transcript was read and reread for recurrent ideas. Codes were assigned to relevant segments of the text; similar codes were aggregated to form themes that were then used to address the research questions and develop coherent narratives [35]. Contradictory data identified during the analysis were initially treated as potential different viewpoints; they were subjected to further analyses to validate or refute them; validated data were used to enrich insights on the issue [36]. To ensure rigor and trustworthiness, transcripts were independently coded, compared and discussed [33]. Respondent validation was ensured by presenting an anonymised summary of the Uganda findings to a community advisory group in Uganda (which included some study participants) with subsequent feedback integrated into the analysis. Emerging findings from the analysis were discussed among the authors in regular qualitative research network meetings.

## Results

A total of 44 IDIs and 15 FGDs were carried out. Participation in the study was high; only 3% of eligible individuals invited failed to participate, with time constraints given as the main reason. Participants characteristics are shown in Table 1. Very few participants had post-secondary education, typical of the population from whom we recruited. Results are presented under seven major themes: awareness and knowledge; attitude towards dolutegravir-based treatment; concerns about side effects; autonomy and choice; contraception availability; preferred ARV attributes; and community engagement. Illustrative quotes from the data are presented in Table 2 and signposted with numbers in squared brackets] in the text.

**Table 1:** Participant characteristics

	South Africa	Uganda
**In-depth interviews**	*N* = 20	*N* = 22
Pregnant	15	9
Lactating woman	5	13
**Age range**		
18–24 years	3	2
> 24 years	17	20
**Education**		
Primary	6	10
Secondary/High school	13	8
Post-secondary	1	2
**Dolutegravir exposure**		
Exposed	8	11
Not exposed	12	11
**Participant type**		
DolPHIN-2 trial participants	8	10
Non-trial participants	12	12
**Focus group discussions**	*N* = 9	*N* = 6
**Gender**		
Women	4 (40 participants)	3 (27 participants)
Men	5 (59 participants)	3 (28 participants)
**Age range**		
18–24 years	18	10
> 24 years	81	45

**Table 2:** key themes in the interviews and focus group discussions

Themes	Illustrative quotes
Awareness and knowledge	**[1]** “*I have heard about it before …*. *[but] I don’t have a lot of information* … they need to explain to us the details. … *what kind of pill it is, how it works and what it does to pregnant women”* (South Africa, Women’s IDI, P191) **[2]** “*They should organise workshops in the community to teach us without discriminating that this one has the virus this one doesn’t have”* (Uganda, Women’s IDI, P205)
Attitude towards dolutegravir-based treatment	**[1] “***dolutegravir is one pill so I am sure you will be able to take it once daily, while the AZTs were two and you were supposed to use it every 4 h. I like it [dolutegravir] because of that*” (South Africa, Women’s IDI, P101). **[2]** *“People lose hope when they are infected with this disease. They say you are dead; you’re finished when you have HIV. But this medicine gives us hope …. It will remove that thought that the tablets are unbearable, that you have HIV... You take it and nobody will know that you have taken it. Nobody will know you are HIV positive.”* (Uganda, Women’s FGD 297, P6). **[3]** “*I take the medicine to help the baby and me, and they say it can affect the baby. I am worried. The old one [efavirenz] affects us a lot [during pregnancy] but I will use it because it doesn’t have that problem [NTDs]. I don’t want anything to affect my baby.*” (Uganda, Women’s IDI, P204) **[4]** “*I would not allow it …. unless we had a child before we became HIV positive. If we don’t have a child, I would not allow it because I still want to have a child. … It doesn’t matter when she uses it, it can still affect the baby. It is better she continues to use the old one [efavirenz].”* (South Africa, Men’s FGD 198, P9) **[5]** “They *explained to me everything [including the risks of NTD], but I said it is ok because the baby was already in danger. I did not book early, and they said the baby may be infected. But with dolutegravir there is a chance …. That is why I took it … to protect the baby …*.” (South Africa, Women’s IDI, P103).
Concerns about side effects	**[1]** “*It is good, because it gives you appetite, you don’t get dizzy. You can still move when you swallow it. But the other ones we had before when you swallow them you have to first stay quiet somewhere. The only problem was that at the beginning I could not sleep well, but it is ok”* (Uganda, Women’s IDI, P295) **[2]** “*It changed my ways especially my female hormones. When I am with a man, I don’t feel it. Sometimes it takes me back to my periods and I get them again …”* (Uganda, Women’s IDI, P208) **[3]** *“Yes [I will accept dolutegravir], as long as it has no negative effect, weakening my body while pregnant, causing me not to eat, remember the child needs to eat.”* (Uganda, Women’s IDI, P201) **[4]** “*They should do proper research this time … they should make sure these side effects we are afraid of will not be there ….”* (South Africa, Women’s FGD 193, P5) **[5]** *“I will not allow it because she would be taking a pill whose side effects, we don’t know …. they should do more research before we allow our wives to use it”* (South Africa, Men’s FGD 198, P2) **[6]** “*I will not like that because I am used to this pill and I don’t know how the other pill will treat me. It took me so long to get use to this one [efavirenz-based regimen].”* (South Africa, Women’s IDI, P 111) **[7]** “*I personally, I would not want to use it now. Because I am afraid that they will make me fat and leave me with no butts and skinny legs.... I would want to see other people use it first because, what if it does something that is irreversible?*” (South Africa, Women’s FGD 192, P4).
Autonomy and choice	**[1]** *“If you force me to use that medicine and I get any problem it means I will put the blame on you so it would be better if we decide for ourselves …. I will feel less pain If I decided to use it and caused problems ….”* (Uganda, Women’s FGD 291, P3). **[2]** ***“****Deciding on their own would be good …. … [But] if one is still giving birth, they should not be given that choice... instead of deciding for themselves, you [health workers] should make the decision for them.”* (South Africa, Men’s FGD 199, P6) **[3]** *“I may decide on my own but I have made the wrong decision and yet it will destroy my health. So, it is good to first consult someone who is better than you … the health workers”* (Uganda, Women’s IDI, P208) **[4]** *“Our lives are in your hands, basawo [health workers]. You know better the side effects. It’s hard for us ordinary people to decide on this …. If you say it is not good for us pregnant women, we will respect that because we know you will make the right decision for us.”* (Uganda, Women’s FGD 292, P5) **[5]** *“the most important thing is educating us more about this drug [dolutegravir]. It’s important to understand about that drug to avoid excuses that the doctor did not educate me that is why I made the wrong decision. If I understand I will make the right decision.”* (Uganda, Women’s IDI, P201) **[6]** *“They should first explain it to us, how it will work and the risks. … they need to explain it first and then ask us to decide. We are capable if they help us.”* (South Africa, Women’s IDI, P191)
Contraception in dolutegravir-based treatment	[**1**] *“Family planning is not a problem for me. I will do it so I can also use the new drug. … family planning will help us not to have infected children”* (South Africa, Women’s IDI, P199) **[2]** ***“****If you give birth every year and you are HIV+, it reduces your health. … it reduces your life span. You need to space the children and go on family planning ….”* (Uganda, Women’s IDI, P207) **[3]** *“It must be given to women who feel like they have had enough children; not the young ones because they still want to get married and have family. … men want children and if she can’t provide that there will be problems ….”* (South Africa, Women’s IDI, P104) **[4]** *“They should give us one that you can easily change so that if you change your mind and you want to have children you can stop.”* (South Africa, women’s IDI, P207) **[5]** “*I feel like the people that are not using contraception, especially the younger ones, will be left behind. It means that if they want to have children, they will not take it.”* (South Africa, women’s IDI, P101). **[6]** *“There is going to be problem because sometimes you find one who is pregnant, and she still goes for family planning. Sometimes the contraceptive will fail. … sometimes it is not the drug, it’s the woman, the thing will expire but she will not go for new one. … sometimes it is the clinic, they keep postponing...” (*Uganda, Women’s FGD 291, P7)
Preferred ARV features	**[1]** “*What I ask [about dolutegravir] is that they should reduce the tablet and confuse it a bit with everyday drugs so that when someone looks at it they do not know it, they don’t notice that you are infected. … it should be a small tablet like Panadol, something that is not different ….”* (Uganda, Women’s FGD 293, P7) **[2]** *“I don’t like it, it makes a lot of noise, it is noisy. It is hard to take it in public. If the new one [dolutegravir] can be made so that when you put it in your bag, it doesn’t make noise.”* (South Africa, Women’s IDI, P113) **[3]** *“I think it [dolutegravir] should be similar to the injection you get from the family planning clinic; you get once monthly. … imagine if you have not told your partner, he will find out if you have three drugs every day”* (South Africa, Women’s FGD 194, P2)
Community engagement	**[1]** “*We need to agree … Me and the mother of the baby …. And see if this thing will not … because there is the thing of one being allergic or not allergic … will this thing be able to protect until … we can rebuild our family* (South Africa, Men’s FGD198, P11).

### Awareness and knowledge

A minority of participants (19 of 42 interviewees) across the two study countries had heard of dolutegravir and the impending transition prior to the study. Most of these were DolPHIN-2 trial participants; others had heard from their peers and social media. No participant reported hearing about dolutegravir on the news, radio or a community programme. They noted similarity with a previous antiretroviral transition in which they relied mostly on informal channels to learn about the new drug.

Nearly all the participants who had heard of dolutegravir said their knowledge of the drug was inadequate and wanted to learn more [1]. Most were aware that dolutegravir is more effective than efavirenz and associated with birth defects when used in pregnancy. Several of them reported inaccurate information, saying dolutegravir: “is an injectable”, “cures HIV”, “causes miscarriage”, “is taken once a month” and “causes tumour in the womb”. As data collection coincided with the release of information on the safety signal, participants reported uncertainty about the safety of dolutegravir. They mentioned the need for more information on risks, conditions of use, contraindications and how to use dolutegravir in pregnancy.

Participants suggested the need for a more inclusive awareness-raising campaign that targets all community members, including HIV positive and negatives. Traditional outreaches that target only PLHIV were deemed to stigmatise and deter people [2]. Although they identified TV, radio and social media (mostly mentioned in South Africa) as important media for awareness campaigns, they worried that they could lead to misinformation and, in the case of social media, exclude rural and uneducated populations. They preferred community-based communication interventions that allow for contact with health professional to address their questions. Additionally, women said they wanted to have more contact time and dialogue with health workers during treatment consultation.

### Attitude towards dolutegravir-based treatment

Most participants had positive views of dolutegravir and thought it was generally more desirable than efavirenz because it provides quicker viral suppression, milder side effects and has low pill burden [1]. Efavirenz-based regimens, with their severe side effects, were perceived to be stigmatising (both internalised and from the community) and incapacitating. They noted that dolutegravir would make HIV patients ‘look and feel normal’ to carry out their daily activities and would encourage ART take up and adherence [2].

However, some participants did have concerns about dolutegravir use in pregnancy. Much of this was related to the risk of NTDs. Following the WHO safety alert, most dolutegravir messages centred around the risk of birth defects, which caused stress and anxiety in the community. Participants were extremely fearful of the risk NTDs and unwilling to risk it for any benefit. Even when it was explained to them that the risk of NTDs was marginal and limited to the first trimester, most (both men and women) were still sceptical. Some women stated that although efavirenz treated them badly during pregnancy (because it made them nauseous, etc) they would rather use it than to risk birth defects with dolutegravir [3]. Men appeared to be less likely to allow their partners to use dolutegravir due to NTDs and a belief that dolutegravir causes infertility among women [4]. Pregnant women who initiated ART late in pregnancy appeared more likely to accept dolutegravir use in pregnancy. Such participants were motivated by the need to minimise the risk of vertical transmission [5]. Women were generally conscious about the lack of evidence on the safety of dolutegravir, being a new drug, and would rather continue to use efavirenz during pregnancy until dolutegravir has been ‘fully tested’ to establish its safety.

There were mixed views on dolutegravir use among women of childbearing potential outside pregnancy. Some participants, mostly men, believed that it was difficult for women to adhere to the conditions for safe usage of dolutegravir (e.g. effective contraception) and suggested that women of childbearing potential should be prevented from using the drug altogether. Most of those who wanted non-pregnant women to use dolutegravir often did not understand that NTDs were caused during conception (and not in pregnancy). They were mainly younger women, from South Africa, and had confidence in their ability to control their fertility plans.

### Concerns about side effects

Most participants who had used dolutegravir reported no side effects, while a few had suffered insomnia, nausea, weight gain and low sex drive [1, 2]. They noted that the effects were relatively less severe compared with previous ARV drugs (e.g. efavirenz) and did not prevent them from taking their pills properly [1]. Even though they worried about side effects, they thought these were acceptable because dolutegravir provides important benefits. Overall, participants noted that it was important for dolutegravir side effects to be mild, particularly for pregnant women to use it as they are already burdened by the effects of pregnancy and other medication. Reflecting on previous ART drugs, they identified “loss of appetite”, “bad dreams”, “dizziness”, “body weakness” and visible symptoms such as rashes as some of the key side effects that could discourage women from using dolutegravir if it were to cause them [3].

Some women, mostly from South Africa, expressed reservations about new ART drugs in general, and dolutegravir by extension, and their potential to cause unforeseen side effects. They worried about not knowing the full range of the side effects of dolutegravir and wanted it to be further “tested” [4, 5]. Some women stated that they would rather “wait-and-see” how it treats other people before deciding to use it [7]. Others lamented over challenges with adjusting to new ART drugs and feared this could discourage some women from switching [6]. In South Africa, concern about weight gain was widely mentioned amongst both male and female participants as a potential barrier to dolutegravir use among women [7]. Only one participant (female) in Uganda mentioned weight gain as a potential issue with dolutegravir, suggesting it may not be as important in that country.

### Autonomy and choice

Participants across both countries agreed on the need for patient involvement in decisions on their ART. There were varied views on whether and how women should be given choice. For some women the desire for choice was based on the need for control over their treatment and fertility plans, to be able to come off dolutegravir and conceive when they liked. Another key point made was that given the seriousness of NTDs it was essential for health workers to protect themselves by transferring the responsibility of decision to women [1]. Some women mentioned that they would feel less resentful if they participated in deciding on their use of dolutegravir that resulted in NTDs. Others stated that giving them autonomy over dolutegravir would enable them take greater responsibility for their treatment and improve treatment adherence [1].

However, most male participants in both contexts and women in Uganda thought it was risky to allow women to decide on their use of dolutegravir, fearing that they would make wrong choices [3]. They believed that health workers were more capable of understanding the relative benefits and risks of dolutegravir and trusted that they would make the best decision for them [4]. Several women in South Africa were confident that, given the necessary information and support, they would be able to make the right decision about their treatment. Women across both countries were receptive to the idea of shared decision-making on dolutegravir involving health workers and patients. They indicated the need for the former to provide information, counselling and support to enable the latter to evaluate available treatment options and chose their preferred treatment [5, 6]. They expressed the need for more information on the benefits and risks of dolutegravir to enable them to make the right decision [6].

### Contraception in dolutegravir-based treatment

Response on the contraception requirement was varied, with older women with children being more receptive to practicing contraception in order to use dolutegravir. Most women living with HIV (WLHIV) were concerned about potential vertical transmission of HIV to their babies and thus welcome the contraception requirement [1]. Some women perceived contraception as an opportunity to space pregnancy and recover their health after the strain of childbirth on their immune system [2].

Most younger women feared that the contraception requirement would hinder fertility plans, strain marriage relations and undermine the prospects of marriage [3]. Some women perceived the contraception requirement as an imposition of family planning on them. Whilst not entirely disapproving of contraception, most women were mainly concerned about the ‘long-acting’ element of the contraception proposed by the WHO guidelines, which was commonly misconstrued to mean permanent contraception. Women expressed a desire for greater control and over their fertility plans, and the need for flexible contraception methods [4]. Another major concern was that the contraception requirement would restrict dolutegravir use among most young women because they desire to have children or lack access to effective contraceptives [5]. In Uganda several women reported serious financial barriers to contraceptive use due to lack of supply in public health facilities and the need for them to purchase from private clinics. Some women also lacked access due to the ARV drug they are using and the associated contraindication with the available contraceptives.

There was scepticism expressed over the extent of protection that contraception could provide against NTDs. A key concern was about contraceptive failure leading to unplanned pregnancies. Participants reported widespread contraception adherence challenges among women across both countries. In Uganda, women reported long delays in the start of a new contraceptive after the expiration of an older one as a potential cause of widespread unplanned pregnancies during dolutegravir use [6].

Contraceptive preference varied between participants. Older women appeared to prefer longer-term contraceptives such as intrauterine device, implant and sterilisation (in the case of South Africa) so that they do not have to visit the facility regularly to renew. Younger women also liked long-acting contraceptives due to the convenience that they offer but preferred those that are not too long term and permanent and are reversible. In South Africa, they desired the contraceptive injection because it was also relatively less invasive, even though they worried that it was being discontinued in the country.

Participants across both countries appreciated the variations in the suitability of different contraceptive methods among women and suggested the need for health workers to assess women to help them identify their most suitable methods. They also wanted women to be actively involved in making decisions about their contraception.

### Preferred ARV attributes

Women identified key drug features that they believe would improve the uptake and adherence to dolutegravir-based treatment when it is introduced. These were rooted in the need to keep their HIV status secret. Reflecting on previous ARV drugs, several women said they wanted dolutegravir pill (e.g. shape, colour and size) to be modified to look like ordinary drugs in the community [1]. They noted that most of the standard ARV drugs were easily identifiable with HIV which discouraged many women from using them if they have not disclosed their status. Women also preferred that the package for dolutegravir is not marked with HIV identifiers; ideally, they should be kept in a plain container and padded to ensure that they do not rattle to draw attention [2]. There was also a desire for injectables and for dolutegravir regimen to be made into a single pill that is taken once daily, weekly or monthly. They thought that reducing the pill burden was essential to decreasing the risk of inadvertent disclosure [3].

### Community engagement

Participants expressed the need for policymakers and programmes to actively engage with WLHIV in order to understand their needs and address them in the rollout. They also noted that the nature of dolutegravir, with its potential teratogenic effects, would require collective decision making between couples for women to use it. Hence, they suggested the need for greater engagement with men to ensure that they are fully informed about dolutegravir. The men also expressed the need to facilitate dialogue between couples over the use of dolutegravir among women [1].

## Discussion

This qualitative study was the first to investigate the acceptability of dolutegravir use in WLHIV in Southern and Eastern Africa. Participants found a dolutegravir-based regimen to be desirable for first line HIV treatment in women except during pregnancy. Concern about the association of dolutegravir with NTDs was the main reason for the reluctance to use dolutegravir in pregnancy. Women were more accepting of dolutegravir use in pregnancy if they are being initiated on ART later in pregnancy. Acceptability was gendered, with nearly all male participants preferring their female spouses of childbearing potential not to use dolutegravir, while most women not planning pregnancy wanted access to contraception alongside dolutegravir. A shared decision-making arrangement involving health workers and patients, with the former providing sufficient information, counselling and support to enable the latter evaluate available treatment options was identified as a fair approach upon which dolutegravir use among women should be determined. Greater dialogue between spouses was recommended to promote acceptability.

There are few previous studies in which to contextualise our findings. Campbell et al. surveyed 636 ART naïve and experienced patients from Uganda and Nigeria who had been initiated on dolutegravir-based regimen in a pilot study [37]. Overall, 90% of the patients reported high acceptability of dolutegravir-based treatment, citing improvement with fewer side effects as the main reason. This level of acceptability appears to be higher than that observed in our study, which may be partly because their study was conducted prior to the NTD signal and participants perspectives may not have been affected by such serious adverse effects. However, Migone and Ghadrshenas carried out a survey with representatives of networks of women living with HIV in sub-Sahara Africa following the signal to examine the acceptability of dolutegravir use in women of childbearing potential [38]. Consistent with our findings, 37% (of 51 respondents) approved and the rest disapproved or were unsure about dolutegravir use among women of childbearing potential. They noted women placed high value on their autonomy and preferred a choice in their ARV regimen. With current evidence suggesting a lower risk of NTDs with periconception use of dolutegravir [13, 14], it will be interesting to see how this may have shifted community attitudes and acceptability of the drug.

The main reasons for the interest in dolutegravir related to its efficacy and safety profile. With its milder side-effects, participants associated dolutegravir with providing greater confidentiality and privacy and mitigating unintended disclosure and internalised stigma. They also noted that it offered hope due to its quick viral suppression feature and will potentially improve ART uptake and adherence. Similar psychosocial benefits have been reported by Kerrigan et al. in relation to patience preference for long-acting ART injectables in US and Spain [39]. However, in South Africa and Uganda, they demonstrate the salience of stigma in the lives of WLHIV and the potential negative role this could have on ART adherence [39–41]. Despite dolutegravir potentially minimising stigma, further structural and biomedical interventions will continue to be needed to accelerate ART uptake and adherence. As noted by the participants, dolutegravir can further contribute to the biomedical solution by strengthening its privacy features with disguised pills appearance and packaging so that they are not easily identifiable with HIV.

Several barriers to optimal acceptability of dolutegravir-based treatment remain. They include concern about dolutegravir related serious adverse events such as NTDs and other ‘unknown risks’ due to it being a new drug. Minor side effects such as insomnia and nausea, while important, are less likely to have significant negative effect on acceptability as there are perceived to be ‘tolerable’ relative to the benefits that dolutegravir offers. Although dolutegravir related weight gain was identified as a potential barrier to acceptability in South Africa, it is unclear what the implications might be in Uganda, and further research may be required. The risk of NTDs was taken extremely seriously among both the men and women participants and remains a significant barrier to uptake of dolutegravir-based treatment. The emerging evidence of a lesser risk of NTDs with dolutegravir could alleviate individuals fears and encourage acceptability but needs to be effectively communicated in communities. Moreover, the lack of contraception services, especially in rural areas, and the financial and logistical burden of contraception could discourage many risk-averse women of childbearing age from initiating on dolutegravir-based treatment. Patient characteristics may also affect acceptability; for example, ART experienced patients who are stable on their regimen expressed less interest for switching to dolutegravir-based treatment as they are unsure about how they will be treated by the potential side effects, and younger women of childbearing potential with no/fewer children were more hesitant towards dolutegravir due to fear of teratogenicity.

The study found limited community awareness and knowledge of dolutegravir-based treatment in South Africa and Uganda. Not only were individuals not aware, there was widespread negative information about dolutegravir which aggravated fears about the drug. Rollout of dolutegravir-based regimen had not started in both countries at the time of the study which may have contributed to the low awareness, even though Uganda was only a few months away. Crucially, the finding demonstrates a weakness in information campaign strategies for new ARV drugs in both countries which, as the participants noted, were mainly facility- and patient- focused; community-based campaigns tend to alienate many community members as they target only HIV positive persons who fail to attend because of stigma. With social networks operating as vital conduits for information and influence on new HIV treatments in resource-limited settings [42, 43], a more inclusive awareness campaign targeting wider community members (including both positives and negatives) is needed to accelerate information dissemination about dolutegravir. Although traditional mass media such as radio and television are widely recognised for their extensive reach, participants expressed preference for community-based communication interventions with contact with health professional to raise awareness and educate about dolutegravir. With widespread negative information and uncertainty about dolutegravir at the community level, individuals longed for dialogue with health professionals in order to learn and seek answers to concerns they have about dolutegravir. Such approach could be useful in alleviating community fears about dolutegravir use in pregnancy. In a study in Philippines, Valido et al. found that the use of community-based communications interventions involving health workers increased community trust and confidence in a new vaccine that had previously been marred in controversy [44].

Consistent with Chadambuka et al. [29], we found that men were very influential in their spouses use of ART and would be crucial to ensuring optimal uptake and adherence to dolutegravir-based treatment among women, particularly due to the potential teratogenicity and contraception. This suggests the need for active engagement with men to encourage women to adopt dolutegravir-based treatment. Existing evidence indicates that male partner involvement does have significant positive impact on ART uptake among their spouses as demonstrated in Zambia [45] and Nairobi [46]. Community interventions promoting male partner involvement in ART need to combine with partner disclosure initiatives to maximise their impact.

This study failed to produce conclusive insights into women’s feelings about a potential dolutegravir related weight gain. Future research needs to investigate the potential association of dolutegravir with weight gain and its implications on acceptability among women. Moreover, it would be interesting to examine the unintended consequences of dolutegravir-based treatment, particularly on early antenatal presentation and ART adherence. With dolutegravir yielding rapid viral suppression, some women may be motivated or discouraged to present early for antenatal care. Research is also needed to explore optimal approaches to health worker-patient communication of ART-related risks and choice.

### Limitations

The study was carried out among pregnant and lactating mothers and their partners, which may limit the generalisation of the results to broader population of WLHIV. Reported acceptability of dolutegravir may have been confounded by some participants involvement in the DolPHIN-2 clinical trial who were given more extensive information and counselling than is normally the case in health facilities. Further, participants were asked to respond to several hypothetical scenarios about dolutegravir use in pregnancy and among women of childbearing potential based on limited information from the WHO 2018 guidelines. Since then new guidelines have been released by the WHO and the Ministries of Health in South Africa and Uganda recommending dolutegravir use in pregnancy and among women of childbearing potential, which may influence attitudes toward the drug. Unlike the DolPHIN-2 trial where participants were given multiple-tablet dolutegravir-based regimen, a fixed dose combination regimen will be used in the national rollout, which may encourage greater acceptability. We could not perform back-translation on the transcripts that were translated from the local languages to English due to resource constraints; however, all the transcripts were checked for accuracy and completeness by the RAs to enhance the validity of the data.

## Conclusions

This study found community desire for dolutegravir use in first line HIV treatment among women due to its potential to enhance health, privacy and reduce HIV-related stigma. However, there is widespread anxiety and reluctance towards dolutegravir use in women during pregnancy due to its association with NTDs. Ensuring optimal community acceptability requires strengthening community awareness and understanding of dolutegravir-based treatment with emphasis on increasing the evidence-base on safety in pregnancy, effective health worker-patient communication of ART related risks, and alleviating concerns about risks in pregnancy. Contraception services need to be improved, including the availability of contraceptive options and integration of contraception and HIV services. It is essential to engage with women to understand their needs and address them in the rollout of dolutegravir, and with men to gain their cooperation and buy-in to women’s use of dolutegravir.

## Supplementary Information

**Additional file 1.**

## Data Availability

Data analysed for this manuscript are available from the corresponding author on reasonable request**.**

## Abbreviations

ART: Antiretroviral therapy
ARV: Antiretroviral
DolPHIN-2: Dolutegravir in Pregnant HIV mothers and Neonates
HIV: Human immunodeficiency virus
IDI: In-depth-interview
LMIC: Low- and middle-income country
NNRTI: Non-nucleoside reverse-transcriptase inhibitor
NTD: Neural tube defect
RA: Research assistant
WLHIV: Women living with HIV
WHO: World Health Organization
