# Supplementary material for: Community acceptability of dolutegravir-based HIV treatment in women: a qualitative study in South Africa and Uganda
Source: BMC Public Health. 2020 Dec 7;20:1883. doi: 10.1186/s12889-020-09991-w (PMC7720619; doi:10.1186/s12889-020-09991-w)
Supplement: Supplementary file 1 — Additional file 1. [file 12889_2020_9991_MOESM1_ESM.doc]

**TOPIC GUIDES**

*Community acceptability of dolutegravir-based HIV treatment in women: a qualitative study in South Africa and Uganda*

1. **IDI with women participants**

**Interview details**

Participant ID #__________ Interviewer Name: _________ Date___________ Audio file #:

Participant type: Pregnant Recent/lactating mother Early Booker Late Booker

DolPHIN-2 trial participant Non-trial participants

**Socio-demographic details**

Age range**:** 18 – 24yrs < 24yrs

Education: Primary or lower Secondary Post-secondary

**Questions**

1. Could you please start by telling me a bit about yourself?

*Probe:*

- Health
- Experience with ART, including ARVs used
- Current and previous pregnancies
- Socio-economic condition

*The government plans to introduce a new HIV drug called Dolutegravir which will replace Efavirenz and other first line drugs. Do you know anything about this? If Yes ask Q2- Q4; If No, skip to Q5*.

1. Tell me about what you know about this drug

Probe:

- Conditions of usage
- Source of information
- Areas where further information is required

1. What do you know about the benefits and risks of dolutegravir?

Probe:

- Benefits and risks in pregnancy
- Benefits and risks in childbearing age
- Personal concerns about dolutegravir

1. Tell me about your experience of using dolutegravir, if you have used it before

*Probe:*

- Reasons/motives for use
- Experience with side effects, if any
- Compare experience with dolutegravir vs efavirenz and other ARVs used previously

1. How do you normally learn/get information about new HIV drugs?

- Probe: What would you like to learn/know about dolutegravir?

1. About this new drug (dolutegravir) that will be introduced, it has been found that it clears the HIV virus more rapidly and has less side effects than most other drugs. But there are suggestions it may be associated with birth defects (neural tube defect) when used at the time of conception and the first trimester. However, it has been found to be safe after the first trimester

*Probe:*

- Would you use it if it is introduced? Why?
- Would you use it if you were planning to fall pregnant? Why?
- Would you use it later in pregnancy (e.g. in the second and third trimesters)? why?

1. When dolutegravir is introduced, women who are likely to fall pregnant [women of reproductive potential] would not be allowed to use it unless they are on ‘adequate’ or ‘effective’ contraception. In other words, a contraceptive method that would prevent the woman from getting pregnant.

*Probe:*

- How do you feel about this condition?
- Would you accept to go on long term contraception in order to use dolutegravir? How?
- Is it possible for women who are on dolutegravir to commit to remain non-pregnant?
- What is an effective form of contraception for you?

1. Tell me about your experience with family planning

*Probe*

- Preferred contraception methods and reasons
- Challenges with contraception
- Challenges with post-partum contraception
- Changes/improvements required

1. Some women have suggested that rather than prevent women of reproductive potential from using dolutegravir they should be given autonomy and choice to decide on whether they want to use dolutegravir or not.

*Probe:*

- How do you feel about this?
- What would you consider as ‘choice’?
- How would you like this to be implemented?

1. What attributes would you like to see included in the dolutegravir regimen when it is introduced?
2. How would you recommend government goes about educating women about dolutegravir?
3. What do you think should be done do ensure greater acceptability and uptake of dolutegravir among women when it is introduced?
4. Is there anything else that you expected us to discuss that we did not?
5. **FGD with women**

**FGD details**

FGD ID #: _________ Facilitator: ________ Notes taker: __________Dates: ____Audio file #:

Gender: _________

**Questions**

1. The government plans to introduce a new HIV drug called Dolutegravir which will replace Efavirenz and other first line drugs. Tell me what you know about this?

*Probe:*

- Conditions of usage
- Source of information about dolutegravir
- Areas where further information is required

1. What are women in your communities saying about dolutegravir?

*Probe:*

- Community perceptions of benefits and risks of dolutegravir?
- Community concerns about dolutegravir

1. Those of you who have used dolutegravir before please tell us about your experience of using the drug.

*Probe:*

- Reasons/motives for use
- Experience with side effects, if any
- Compare experience with dolutegravir vs efavirenz and other previous ARVs

1. About this new drug (dolutegravir) that will be introduced, it has been found that it clears the HIV virus more rapidly and has less side effects than most other drugs. But there are suggestions it may be associated with birth defects (neural tube defect) when used at the time of conception and the first trimester. However, it has been found to be safe after the first trimester.

- *Probe:* Acceptability among women of use in pregnancy and later in pregnancy (e.g. in the second and third trimesters)

1. When dolutegravir is introduced, women who are likely to fall pregnant [women of reproductive potential] would not be allowed to use it unless they are on ‘adequate’ or ‘effective’ contraception. In other words, a contraceptive method that would prevent the woman from getting pregnant.

*Probe:*

- Acceptability of the contraception requirement
- Barriers and challenges to the contraception condition
- Perception of effective form of contraception

1. Please share with us your experiences with family planning

*Probe:*

- Knowledge of birth control options for HIV positive women?
- Preferred contraception methods and reasons
- Challenges with contraception
- Challenges with post-partum contraception
- Changes/improvements required

1. Some women have suggested that rather than prevent women of reproductive potential from using dolutegravir they should be given autonomy and choice to decide on whether they want to use dolutegravir or not.

*Probe:*

- How do you feel about that?
- What do you consider as ‘choice’?

1. What attributes would you like to be included in the dolutegravir regimen when it is introduced?
2. How do women in this community normally learn/get information about new HIV drugs?

- *Probe:* How should government raise awareness or educate people about dolutegravir?

1. What do you think are likely to be the main challenges to women accepting to use dolutegravir when it is introduced?

- *Probe:* What would make women accept it?

1. Is there anything else that you expected us to discuss that we did not?

1. **FGD with male partners**

**FGD details**

FGD ID #: _________ Facilitator: ________ Notes taker: __________Dates: ____Audio file #:

Gender: _________

**Questions**

1. The government plans to introduce a new HIV drug called Dolutegravir which will replace Efavirenz and other first line drugs. Tell me what you know about this?

*Probe*

- Conditions of usage
- Source of information about dolutegravir
- Areas where further information is required
- Benefits and risks

1. About this new drug (dolutegravir) that will be introduced, it has been found that it clears the HIV virus more rapidly and has less side effects than most other drugs. But there are suggestions it may be associated with birth defects (neural tube defect) when used at the time of conception and the first trimester. However, it has been found to be safe after the first trimester.

Probe:

- Would you allow your partners to use dolutegravir if she was planning to fall pregnant? Why?
- Would you allow your partners to use dolutegravir after the first trimester of pregnancy when the drug is purported to be safe? Why?
- Concerns about women using dolutegravir

1. When dolutegravir is introduced, women who are likely to fall pregnant [women of reproductive potential] would not be allowed to use it unless they are on ‘adequate’ or ‘effective’ contraception. In other words, a contraceptive method that would prevent the woman from getting pregnant.

*Probe:*

- Would you accept to practice contraception with your spouse so that she can use dolutegravir?
- Barriers and challenges to the contraception condition
- Perception of effective form of contraception

1. What are the main challenges that are likely to affect women’s uptake of dolutegravir when it is introduced?

- *Probe:* What would make women accept it?

1. What would make men to accept for their spouses to use dolutegravir when it is introduced?
2. Is there anything else that you expected us to discuss that we did not?
